# Autistic Symptoms in Schizophrenia Spectrum Disorders: A Systematic Review and Meta-Analysis

**DOI:** 10.3389/fpsyt.2019.00078

**Published:** 2019-02-21

**Authors:** Franco De Crescenzo, Valentina Postorino, Martina Siracusano, Assia Riccioni, Marco Armando, Paolo Curatolo, Luigi Mazzone

**Affiliations:** ^1^Department of Psychiatry, University of Oxford, Oxford, United Kingdom; ^2^Pediatric University Hospital-Department, Bambino Gesù Children's Hospital, Rome, Italy; ^3^Department of Epidemiology, Lazio Regional Health Service, Rome, Italy; ^4^Department of Pediatrics, School of Medicine, University of Colorado Anschutz Medical Campus, JFK, Aurora, CO, United States; ^5^Brain and Body Integration - Mental Health Clinic, Denver, CO, United States; ^6^Department of Biomedicine and Prevention, University of Rome Tor Vergata, Rome, Italy; ^7^Department of Biotechnological and Applied Clinical Sciences, University of L'Aquila, L'Aquila, Italy; ^8^Child Neurology and Psychiatry Unit, System Medicine Department, University of Rome Tor Vergata, Rome, Italy; ^9^Developmental Imaging and Psychopathology Lab, Department of Psychiatry, School of Medicine, University of Geneva, Geneva, Switzerland

**Keywords:** schizophrenia, autism, development, comorbidity, meta-analysis

## Abstract

**Background:** Recent studies have examined the association between autism spectrum disorder and schizophrenia spectrum disorders, describing a number of cognitive features common to both conditions (e.g., weak central coherence, difficulties in set-shifting, impairment in theory of mind). Several studies have reported high levels of autistic symptoms in population with schizophrenia spectrum disorders. Our study systematically reviews and quantitatively synthetizes the current evidence on the presence of autistic symptoms in individuals with schizophrenia spectrum disorders.

**Methods:** A comprehensive literature search of the PubMed/MEDLINE, Cochrane Library, CINHAL, and Embase databases was performed from the date of their inceptions until March 2018. The primary outcome measure was the Autism Spectrum Quotient (AQ). As secondary outcome measures, we analyzed the AQ subscales. Data were extracted and analyzed by using a conservative model and expressed by standardized mean difference (SMD).

**Results:** Thirteen studies comprising a total of 1,958 individuals were included in the analysis. Results showed that individuals with schizophrenia spectrum disorders have higher levels of autistic symptoms compared to healthy controls [SMD: 1.39, 95% confidence interval (CI): 1.11 to 1.68] and lower levels of autistic symptoms compared to individuals with autism (SMD: −1.27, 95% CI: −1.77 to −0.76).

**Conclusions:** Current findings support that individuals with schizophrenia spectrum disorders have higher autistic symptoms than healthy controls. Therefore, further studies are needed in order to shed light on the association between these two conditions.

## Introduction

According to the current diagnostic classification systems, Schizophrenia Spectrum Disorders (SSDs) include schizophrenia, schizophreniform disorder, and other psychotic disorders (1). These disorders are defined by abnormalities in one or more of the following five domains: delusions, hallucinations, disorganized thinking (speech), grossly disorganized or abnormal motor behavior (including catatonia), and negative symptoms. The onset of schizophrenia spectrum disorders is usually between late teens and mid-30s. Onset prior to adolescence is rare (2).

By contrast, Autism Spectrum Disorder (ASD) is an early onset lifelong condition characterized by persistent deficits in social communication, as well as restricted and repetitive patterns of behavior (3). Until a century ago, autism was considered as an early expression of schizophrenia. Later on, different studies have demonstrated that these conditions are separate (3–5). Despite their differences, SSDs and ASD appear to show several similar symptoms and research into the possible link between these disorders has grown considerably with studies showing shared genetic risk factors as well as potential links in specific clinical characteristics of these two disorders [for a systematic review see Kincaid et al. (6)]. Recent studies have shown a genetic overlap between ASD and SSDs (7). For example, studies assessing copy number variation (CNV) in ASD and SSDs have repeatedly observed heterozygous deletions eliminating exons of the neurexin-1α gene (but not the neurexin-1β gene) in patients with ASD and SSDs (8–10). Furthermore, several studies have reported high number of shared CNV deletions and duplications, including 1q21, 15q11.2, 15q13.3, 16p11.2, 22q12, and Neurexin 1 loci, in ASD and SSDs (8, 11).

Moreover, an overlap between early autistic symptoms and psychotic experiences during adolescence was reported in longitudinal studies reporting that 20–50% of individuals with childhood-onset schizophrenia met criteria for premorbid ASD (12–16). In addition, social difficulties and language impairment are common to both conditions. Specifically, deficits in reciprocal social interactions are considered part of the core clinical symptoms of ASD. In fact, individuals with ASD exhibit deficits in eye contact, non-verbal communication (e.g., descriptive, conventional, and emphatic gestures), and difficulties to develop age-appropriate relationships (17). Similarly, social withdrawal is documented in individuals with SSDs. Indeed, impairment in social functioning common to both conditions may be due to underlying mechanisms (e.g., deficits in theory of mind) that are common to both conditions (18–24). Studies investigating social functioning deficits in these conditions have shown contrasting findings. For example, Couture et al. (23) completed a battery of social cognitive measures in 44 individuals with schizophrenia, 36 individuals with high functioning autism, and 41 non-clinical controls and reported that individuals with schizophrenia and individual with high functioning autism were both impaired on a variety of social cognitive tasks. By contrast, Sasson et al. (24) comparing the visual scanning patterns and emotion judgments of individuals with autism, individuals with schizophrenia, and controls, suggested that both individuals with autism and individuals with schizophrenia fixate faces less than controls. However, their results also found that only individuals with autism fail to orient to faces more rapidly based on the presence of facial information (24).

In clinical practice, distinguishing between these conditions has proved to be challenging given the symptom overlap between ASD and SSDs. For example, social communication deficits and restricted and repetitive behaviors typical of ASD can be misinterpreted as possible signs of a SSD (25). Some perceptions reported by individuals with ASD are misinterpreted as hallucinations (26). Deficits in emotion recognition leading to misinterpretations of the actions of others is a core symptom of ASD and is also common in SSDs (26). Moreover, difficulties with emotional reciprocity or speech delay in ASD can be misinterpreted as blunted affect or alogia (poverty of speech) in SSDs (26). Furthermore, catatonic features are present in both disorders (26, 27). Previous systematic reviews suggested elevated rates of co-occurrence of ASD and SSDs (28, 29). For example, Kincaid et al. (6) in a recent systematic review reported a prevalence rate of ASD that ranged from <1 to 52% across outpatient and inpatient populations with a diagnosis of schizophrenia or other psychotic disorders. However, considering ASD as dimensional disorder, separate from the issue of ASD diagnosis is the matter of autistic traits. Autistic traits refer to symptoms that are typical of ASD at the time of the assessment; however, these symptoms are generally not reported during childhood, which is essential for an ASD diagnosis (6).

Studying autistic symptoms in individuals with SSDs can give further insight to understand the overlap and distinction between these conditions, which can have important diagnostic and treatment implications (22). For example, previous studies reported that individuals with SSDs with autistic features had a longer duration of illness compared to individuals with SSDs without autistic symptoms (30, 31). Moreover, these studies also suggest that a longer duration of illness is associated with poorer long-term outcomes and a higher symptom severity in this clinical population (32). Therefore, an early screening of autistic symptoms in individuals with SSDs might be able to inform both psychological and pharmacological treatments, and possibly modify the clinical outcome in this clinical population (6). Kincaid et al. (6) systematic review reported prevalence rates of autistic symptoms across outpatient and inpatient populations with a diagnosis of schizophrenia or other psychotic disorders ranging from 9.6 to 61%. However, to our knowledge, no previous meta-analysis was conducted in order to quantify the presence of autistic symptoms in SSD populations. Therefore, the aim of this systematic review and meta-analysis was to systematically review and quantitatively synthetize the current evidence on the presence of autistic symptoms in individuals with SSDs compared to healthy controls and individuals with autism.

## Methods

### Literature Search

**Figure 1:**
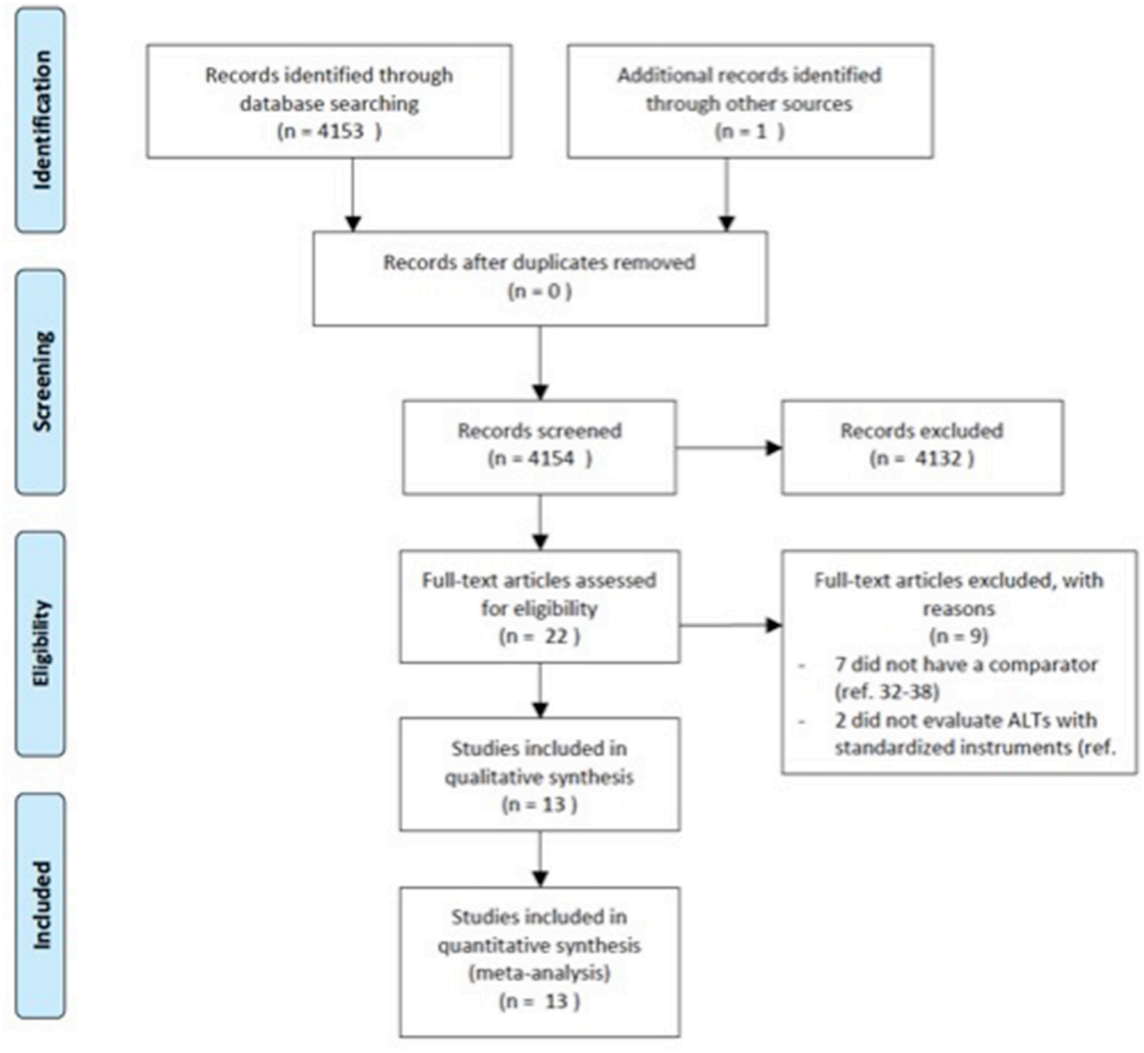
PRISMA flow diagram.

The electronic databases of PubMed, Medline, CINAHL, ISI web of knowledge were searched up from the date of their inceptions until March 2018. We used a search algorithm based on a combination of the terms: autist^*^, “autistic disorder,” “autism,” “child development disorders, pervasive,” “Asperger syndrome,” and “schizophrenia,” “schizophrenia spectrum and other psychotic disorders,” “schizophrenia, paranoid,” “schizophrenia, disorganized,” “schizophrenia, childhood,” “schizotypal personality disorder.” Reference lists of eligible papers were also screened for relevant studies. No language limit was used. We followed the Preferred Reporting Items for Systematic Reviews and Meta-Analyses (PRISMA) guideline (33) (Figure 1).

### Study Selection

All studies using recognized assessment scales to measure autistic symptoms in individuals with SSDs, compared to healthy controls or autism, were eligible for inclusion. We included study-defined diagnosis of first-episode psychosis or SSDs [i.e., schizophrenia, psychotic disorder not otherwise specified (NOS), schizoaffective disorder, schizophreniform disorder, delusional disorder]. The exclusion criteria were: (a) articles not within the field of interest of this review; (b) review articles, editorials, or letters, comments, conference proceedings; (c) case reports or case series; (d) studies dated before 1990 if the system used for the diagnosis of schizophrenia did not use operationalized criteria, but only disease names with no diagnostic criteria (i.e., ICD-9); (e) We excluded mood disorders with psychotic features (e.g., major depression with psychotic symptoms, bipolar disorder with psychotic symptoms). Two researchers (MS, VP) independently identified potential titles from all databases and screened the abstracts for relevance. Full-texts were then retrieved and read to determine eligibility. Disagreements were resolved by consensus.

### Data Extraction

For each included study, the same two reviewers independently documented information about the publication (i.e., author's names, journal, year of publication, setting), patients' and comparison's characteristics (i.e., gender, age, diagnostic criteria, outcomes). We assessed the quality and potential sources of bias for each study by using the Newcastle Ottawa scale (NOS) (34) (Table 1).

**Table 1:** Risk of bias table for assessing the quality of cohort studies by using the Newcastle-Ottawa Scale.

**Study**	**Representativenes of the exposed cohort**	**Selection of the non-exposed cohort**	**Ascertainment of exposure**	**Demonstration that outcome of interest was not present at start of study**	**Comparability of cohorts on the basis of the gender**	**Comparability of cohorts on the basis of the age**	**Assessment of outcome**	**Was follow-up long enough for outcomes to occur**	**Adequacy of follow up of cohorts**
Konstantareas et al. (35)	^*^	^*^	^*^		^*^	^*^	^*^	^*^	^*^
Naito et al. (36)	^*^	^*^	^*^		^*^			^*^	^*^
Sasamoto et al. (37)	^*^		^*^		^*^	^*^		^*^	^*^
Solomon et al. (18)		^*^	^*^		^*^	^*^			^*^
Guo et al. (38)		^*^	^*^						^*^
Jalbrzikowski et al. (39)		^*^	^*^		^*^	^*^			^*^
Lugnegard et al. (40)	^*^	^*^	^*^		^*^			^*^	^*^
de Bildt et al. (41)	^*^	^*^	^*^		^*^	^*^	^*^	^*^	^*^
Matsuo et al. (42)	^*^	^*^	^*^		^*^			^*^	^*^
Martinez et al. (43)	^*^	^*^	^*^		^*^	^*^		^*^	^*^
Zhang et al. (44)	^*^	^*^	^*^		^*^	^*^		^*^	^*^
Ota et al. (45)	^*^		^*^					^*^	^*^
Upthegrove et al. (46)			^*^		^*^				^*^

* *item present. i.e., low risk of bias for for the variable considered*.

### Outcome Measures

The Autism Questionnaire (AQ), expressed as continuous variable was used as the primary outcome measure. AQ subscales were used as secondary outcome measures. The AQ is a 50-item questionnaire consisting of five different areas: social skill, attention switching, attention to detail, communication, and imagination (47). Whenever the AQ was not measured or reported, the following scales measuring symptoms of autism were used: the Social Responsiveness Scale (SRS) (48), the Autism Diagnostic Observation Schedule-Second Edition (ADOS-2) (49, 50), the Autism Spectrum Screening Questionnaire (ASSQ) (51), and the Childhood Autism Rating Scale (CARS) (52, 53).

The SRS is a 65-item rating scale focusing on social awareness and avoidance (54, 55).

The ADOS-2 is a semi-structured, standardized assessment of communication, social interaction, play, and repetitive behaviors. The ADOS-2 includes five modules, The choice of the module is based on the level of expressive language and chronological age of the individual being evaluated (i.e., Module Toddler: from 12 to 30 months of age who do not consistently use phrase speech; Module 1: children 31 months and older who do not consistently use phrase speech; Module 2: children of any age who use phrase speech, but are not verbally fluent; Module 3: verbally fluent children or adolescents; Module 4: verbally fluent older adolescents and adults) (49, 50).

The ASSQ is a teacher's or parent's rating scale that investigates four main fields: social difficulties, tic/motor/obsessive-compulsive disorders, and autistic style (51).

The CARS is a clinician-rated observation scale evaluating socialization, communication, emotional responses, and sensory sensitivities. This scale has shown good reliability, a high degree of correlation with the Diagnostic and Statistical Manual of Mental Disorders (DSM)- Fourth Edition-Text Revised criteria, and a good agreement with diagnoses made using the ADOS-2 and the Autism Diagnostic Interview-Revised (ADI-R) (56).

### Data Analysis

We synthesized study included in the current review consistently with meta-analytic recommendations (57). Data analysis were performed using STATA (version 13.1). data analysis involved the following steps: (a) calculating standardized mean difference (SMD) for each comparison with confidence intervals (CI) (95%); (b) determining an overall average SMD; (c) estimating heterogeneity. We considered SMD “small” if <0.40, “moderate” from 0.40 to 0.70, and “large” if >0.7 (58). The combined use of slightly different scales suggested application of the random effects model, which is more conservative than the fixed-effects model. Qualitative data were presented descriptively. The *I*^2^ statistic was used to assess the heterogeneity of effect sizes (57). The *I*^2^ statistic rages from 0 to 100 and measures the percent of variation across effect sizes due to heterogeneity compared to chance. A high *I*^2^ index indicates greater heterogeneity and greater variation in effect size across studies. We used *I*^2^ thresholds of 25, 50, and 75% to differentiate low, moderate and high heterogeneity. We also undertook subgroup analyses for studies on adults, = studies on children, studies on adolescents, studies including only patients diagnosed with schizophrenia, studies including also individuals diagnosed with SSDs, and studies including also individuals with an unspecified psychotic episode.

## Results

### Selected Studies

The literature search generated 4,153 articles. 4,131 articles were excluded due to the fact that they did not meet the inclusion criteria. Twenty-two articles were screened for eligibility by full-text review. Of these, seven did not have a comparator group (32, 59–64) and two did not evaluate autistic symptoms with standardized instruments (65, 66). A total of 13 studies comprising 1,958 individuals were included in the current meta-analysis.

### Study Characteristics

The characteristics of the included studies are presented in Table 2. The study population mean age was 25.4 years. Three studies included only children and adolescents (18, 38, 39). Four studies were undertaken in Japan (36, 37, 42, 45), four in Europe (35, 40, 41, 46), three in the U.S. (18, 39, 43), and two in China (38, 44). Most of the studies included a high percentage of males, with the exception of three studies (40, 42, 45). Most of the studies included patients with a diagnosis of schizophrenia. Nine studies included a healthy control group, one study included first degree relatives as control group (41), and eight studies included a group with a diagnosis of autism. Seven studies assessed the presence of autistic symptoms in other clinical groups (i.e., psychopathy, attention-deficit/hyperactivity disorder, ultra-high risk (UHR) to develop psychosis, first episode psychosis, obsessive compulsive disorder, major depressive disorder, bipolar disorder) (18, 38, 39, 41, 42, 44, 46). To support the diagnosis, one study used the DSM-Third Edition-Text Revised criteria (35), one study used the DSM-Fourth Edition criteria (38), four studies used the DSM-Fourth Edition-Text Revised criteria (18, 36, 39, 44), and one study used the DSM-Fifth Edition criteria in one study (45). The quality assessment is described in Table 1. Of note, we used self-reported scales which may introduce a bias for the assessment of the outcome.

**Table 2:** Included studies.

**Studies**	**Year**	**Setting**	**Participants (n)**	**Diagnostic criteria**	**Mean Age (SD)**	**Gender (% males)**	**Outcomes**	**Outcome Measures Total Score Mean (SD)**
Konstantareas et al. (35)	2001	Continuing care program of a psychiatric research institute	Autism (14) Schizophrenia (14)	DSM-III-; Leiter; SANS; SAPS; SCID.	25.3 (4.4) 25.3 (4.4)	100 100	CARS	35.36 (4.4) 19.36 (2.1)
Naito et al. (36)	2010	Kobe university hospital Kansai-Seishonen Sanatorium	Autism (51) Schizophrenia (46)	DSM-IV-TR; S-scale; WAIS-R; WAIS-III.	28.8 (9.4) 34.1 (9.6)	78 50	AQ Adult Japanese Version	32.6 (6.8) 21.8 (7.4)
Sasamoto et al. (37)	2011	Department of Neuropsychiatry, Kyoto University Hospital	Schizophrenia (20) Healthy controls (25)	JART; PANSS; SCID-I/P (version 2.0); WAIS–R (vocabulary and block design).	34.5 (8.8) 34.5 (9.4)	70 64	AQ Adult Japanese Version	25.35 (6.6) 14.48 (6.9)
Solomon et al. (18)	2011	UC Davis EDAPT clinic	Clinical High risk for psychosis (met criteria for the attenuated positive symptom risk state) (15) First episode schizophrenia (16) Autism (20) Healthy controls (20)	ADOS-G mod 3, 4; CCC-2; DSM-IV-TR; SCID-I/P; SCQ; SIPS; WASI.	14.1 (2.2) 17.0 (1.8) 15.1 (2.2) 14.8 (2.1)	66 75 80 65	SRS	(13.9)72.5 (20.7) 79.55 (11.8) 42.8 (9.3)
Guo et al. (38)	2011	Institute of mental health Peking University	Autism (94) ADHD (45) Childhood onset schizophrenia (26) Healthy controls (120)	DSM-IV	6.7 (3.9) 8.8 (2.2) 13.8 (3) 6.0 (1.3)	Not Reported	CH-ASSQ Mandarine Chinese Version	25.3 (9.2) 10.4 (7.1) 12.2 (10.6) 25.3 (9.2)
Jalbrzikowski et al. (39)	2013	CAPPS or the adolescent brain and behavior reaserch center	Youths at clinical high risk (58) Adolescents with a psychotic disorder (20) Healthy controls (36)	DSM-IV-TR; K-SADS; SCID-I/P; SIPS; WASI.	15.5 (1.9) 15.7 (1.6) 15.0 (1.5)	64 45 50	GFS; GFR; aSRS	67.2 (15) 70.7 (12.2) 47.8 (11.4)
Lugnegard et al. (40)	2014	Department of Psychiatry Central Hospital Karlstad Sweden	Asperger Syndrome (51) Schizophrenic psychosis (schizophrenia, schizoaffective disorder, schizophreniform disorder) (36) Healthy controls (49)	DISCO-11; SCID-I; WAIS-III (vocabulary)	27.1 (4.1) 29.1 (4.2) 28.6 (9.2)	47 63 38	AQ Adult Version	26.7 (8.9) 22.7 (6.2) 13.4 (6.4)
de Bildt et al. (41)	2015	University Medical Center Groningen	ASD-high functioning (38) Schizophrenia (18) Psychopathy (16) Controls (first degree relatives) (21)	DSM-IV-TR	31.8 (11.2) 37.0 (10.7) 39.0 (10.6) 34.2 (9.1)	100 100 100 100	ADOS-2 module 4 revised algorithm (Hus and Lord 2014)	10.37 (5.7) 7.28 (4.1) 3.31 (2) 2.67 (2.3)
Matsuo et al. (42)	2015	National Center of Neurology and Psychiatry Hospital, Tokyo	Schizophrenia (44) Major depressive disorder (125) Bipolar disorder (56) Healthy controls (65)	DSM-IV-TR; HDRS-17; Japanese version of Mini International Neuropsychiatric Interview; PANSS; WAIS-III; YMRS.	36.9 (7.5) 41.5 (9.2) 40.4 (7.8) 42.2 (8.2)	46 56 46 28	SRS for Adults	59.6 (25) 48.7 (25) 55.4 (25.8) 32.5 (19.1)
Martinez et al. (43)	2016	Service Hospitalo-Universitaire in Sainte-Anne Hospital, Paris	Schizophrenia (36) Autism (19) Healthy controls (20)	ADI-R; BPRS-24; DIGS; DSM-IV-TR; MASC-FR; PANSS; TLC; WAIS III.	23.4 (3.5) 22.7 (4.1) 23.4 (3.6)	83 78 85	AQ	20.9 (7.5) 30.2 (8.3) 14.1 (10.1)
Zhang et al. (44)	2016	Mental Health Center of Anhui Province	Autism (32) Schizophrenia (37) Obsessive-compulsive disorder (38) Healthy controls (38)	DSM IV-TR; Raven test.	19.4 (3.8) 20.9 (3.6) 21.2 (3.1) 21.3 (3.3)	81 81 81 78	AQ Mandarin Chinese Version	133.4 (10.1) 120.5 (6.8) 118.3 (8.3) 103.5 (8.5)
Ota et al. (45)	2017	National Center of Neurology and Psychiatry Hospital, Tokyo	Schizophrenia (37) Healthy controls (62)	DSM-5; Japanese Mini-International Neuropsychiatric Interview; PANSS.	36.2 (9.5) 40.6 (13.)	45 27	SRS for Adults	60.6 (24) 32.2 (16)
Upthegrove et al. (46)	2017	Birmingham Early Intervention Services	First episode psychosis (99) Healthy controls (381)	AQ; BHS; CAPEp; CESD-R; PANSS; SBQ-R.	25.6 (5.0) 20.6 (3.0)	67 21	AQ	20.76 (8) 15.32 (6)

*ADOS-G, Autism Diagnostic Observation Schedule, Generic; ADOS-2, Autism Diagnostic Observation Schedule, Second Edition; ADI-R, Autism Diagnostic Interview – Revised; AQ, Autism-Spectrum Quotient; BHS, Beck Hopelessness Scale; BPRS, Brief Psychiatric Rating Scale; CAPEp, Community Assessment of Psychotic Experiences; CARS, Childhood Autism rating Scale; CCC-2, Children's Communication Checklist-2; CESD-R, The Center for Epidemiologic Studies Depression Scale-Revised; CH-ASSQ, Autism Spectrum Screening Questionnaire; DIGS, Diagnosis Interview for Genetic Studies; DISCO 11, The diagnostic interview for Social and Communication Disorders version 11; DSM III-R, Diagnostic and Statistical Manual of Mental Disorders, Third Edition, Revision; DSM-IV-TR, Diagnostic and Statistical Manual of Mental Disorders, Fourth Edition, Text Revision; DSM-5, Diagnostic and Statistical Manual of Mental Disorders, Fifth Edition; JART, Japanese version of the National Adult Reading Test; K-SADS, Kiddie Schedule for Affective Disorders and Schizophrenia; HDRS-17, Hamilton Depression Rating Scale; LEITER, Leiter International Performance Scale; MASC-FR, Movie for the Assessment of Social Cognition; PANSS, Positive and Negative Syndrome Scale; SANS, Scale for the Assessment of Negative Symptoms; SAPS, Scale for the Assessment of Positive Symptoms; SBQ-R, The Suicide Behaviours Questionnaire-Revised; S-scale, Additional questions about psychotic symptoms; SCID-I/P, Structured Clinical Interview for DSM-IV-TR; SCQ, Social Communication Questionnaire; SIPS, Structured Interview for Prodromal Symptoms; SRS, Social Responsiveness Scale; TLC, Thought Language and Communication Scale; WAIS, Wechsler Adult Intelligence Scale; WASI, Wechsler Abbreviated Scale of Intelligence; YMRS, YoungMania Rating Scale*.

### Meta-Analysis

**Figure 2:**
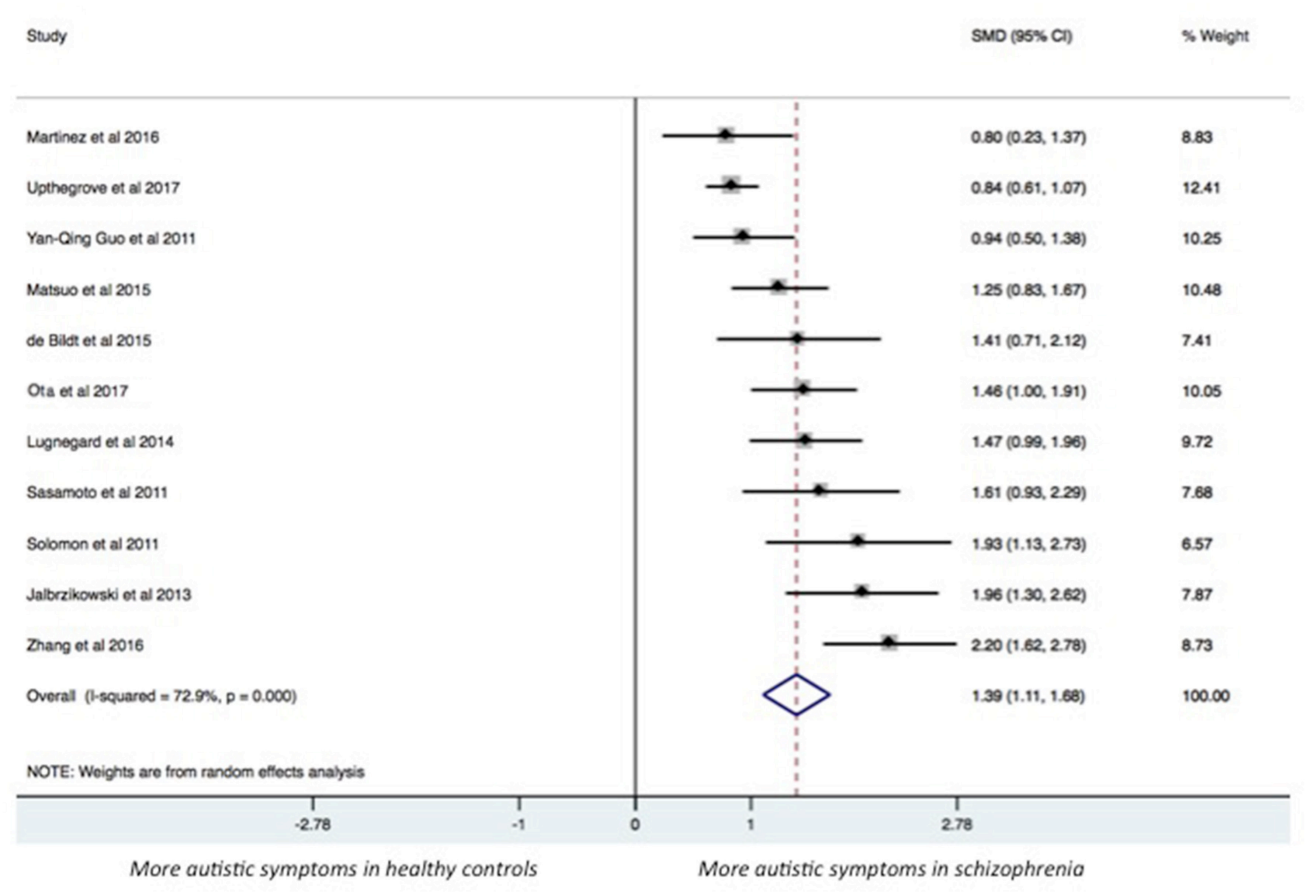
Forest plot.

The results of the pairwise meta-analysis for individuals with SSDs vs. healthy controls and individuals with autism compared for the presence of autistic symptoms in our study are presented in Table 3 (See Forest Plot, Figure 2).

**Table 3:** Data analysis results.

**Comparison**	**Outcome**	**Studies**	**SMD**	**95% CI**	**z-score**	***p*-value**	**I^**2**^**
**Schizophrenia vs. healthy controls**							
	all scales	11	1.39	1.11 to 1.68	9.50	0.000	72.9%
	AQ total	5	1.68	0.84 to 2.51	3.93	0.000	93.0%
	AQ social	3	1.24	0.94 to 1.54	8.08	0.000	0.0%
	AQ switching	3	0.75	0.03 to 1.47	2.04	0.042	83.4%
	AQ attention to details	3	0.27	−0.01 to 0.54	1.90	0.057	0.0%
	AQ communication	3	1.38	0.99 to 1.77	7.01	0.000	35.2%
	AQ immagination	3	0.96	0.67 to 1.25	6.45	0.000	0.0%
**Schizophrenia vs. autism**							
	all scales	8	−1.27	−1.77 to−0.76	4.95	0.000	84.0%
	AQ total	4	−1.17	−1.69 to−0.65	4.43	0.000	76.9%
	AQ social	3	−0.66	−1.09 to−0.23	3.05	0.002	64.2%
	AQ switching	3	−0.87	−1.13 to−0.61	6.59	0.000	0.0%
	AQ attention to details	3	−0.59	−0.84 to−0.34	4.59	0.000	0.0%
	AQ communication	3	−0.70	−1.38 to−0.02	2.02	0.043	85.4%
	AQ immagination	3	−0.56	−1.07 to−0.06	2.02	0.028	74.5%
**SUBGROUP ANALYSES**							
**Schizophrenia vs. healthy controls**							
	Age > 18 y, all scales	8	1.35	1.02 to 1.68	8.05	0.000	74.3%
	Age < 18 y, all scales	3	1.56	0.81 to 2.31	4.09	0.000	76.9%
	Only schizophrenia, all scales	8	1.27	0.96 to 1.59	7.84	0.000	73.4%
	Schizophrenia spectrum, all scales	3	1.70	1.35 to 2.05	9.48	0.000	0.0%
**Schizophrenia vs. autism**							
	Age > 18 y, all scales	5	−1.06	−1.52 to −0.60	4.54	0.000	75.1%
	Age < 18 y, all scales	3	−1.96	−3.54 to −0.38	2.44	0.015	92.5%
	Only schizophrenia, all scales	1	−2.80	−3.36 to −2.24	9.75	0.000	0.0%
	Schizophrenia spectrum, all scales	2	−0.48	−0.85 to −0.12	2.61	0.009	0.0%

Individuals with SSDs have significantly higher autistic symptoms than healthy controls (SMD: 1.39; 95% CI: 1.11 to 1.68) and lower autistic symptoms than individuals with autism (SMD: −1.27; 95% CI: −1.77 to −0.76). Individuals with SSDs have significantly higher autistic symptoms than healthy controls on the AQ total (SMD: 1.68; 95% CI: 0.84 to 2.51), the AQ Social subscale (SMD: 1.24; 95% CI: 0.94 to 1.54), the AQ Attention Switching subscale (SMD: 0.75; 95% CI: 0.03 to 1.47), the AQ Communication subscale (SMD: 1.38; 95% CI: 0.99 to 1.77), and the AQ Imagination subscale (SMD: 0.96; 95% CI: 0.67 to 1.25). However, no significant difference was found between individuals with SSDs and healthy controls on the AQ Attention to Details subscale (SMD: 0.27; 95% CI: −0.01 to 0.54).

Individuals with SSDs have significantly lower autistic symptoms than individuals with autism on the AQ total (SMD: −1.17; 95% CI: −1.69 to−0.65) and on all the AQ subscales (see Table 3).

We undertook subgroup analyses on studies on children, on studies on adolescents, and on studies reporting a diagnosis of SSD, and we found that results did not substantially change (see Table 3).

## Discussion

The aim of this meta-analysis was to provide a synthesis of existing literature on the presence of autistic symptoms in individuals with SSDs. The meta-analysis results show that individuals with SSDs have significantly higher autistic symptoms than healthy controls and lower autistic symptoms than individuals with autism. The results of this meta-analysis support a shared symptomatology between these conditions. For example, language deficits often found in individuals with autism are also frequently found in prodromal symptoms of SSDs and were highlighted by the difference on the AQ Communication subscale between individuals with SSDs and healthy controls (SMD: 1.38) (67). Moreover, tangential thought, formal language, and focus on favorite subjects are often present in individuals with autism, and these language symptoms are similar to formal thought disorder which is characterized by disorganized speech (22). Furthermore, individuals with autism may present with language deficits characterized by a lack of verbal initiation and poverty of content similar to individuals with schizophrenia and negative symptoms (22). Likewise, social deficits seem to be present in both conditions (18, 22). For example, social isolation and difficulties to maintain age-appropriate peer relationships are observed in individuals with autism as well as in individuals with schizophrenia. Therefore, it is not surprising that the AQ Communication subscale, which measure the communication skills, and the AQ Socialization subscale, which measure the socialization ability, reported that highest scores in this group. On the other hand, it has to be noted that the level of autistic symptoms changes among the AQ subscales. Indeed, the difference in the AQ sub-scale scores between individuals with SSDs and healthy controls showed higher levels of autistic symptoms in individuals with SSDs on the AQ Communication subscale (SMD: 1.38), on the AQ Social subscale (SMD: 1.24), the AQ Imagination subscale (SMD: 0.96), and the AQ Attention Switching subscale (SMD: 0.75). Notably, no significant difference was found between individuals with SSDs and healthy controls on the AQ Attention to Details subscale (SMD: 0.27), thus suggesting that in this clinical population the difficulties on some autistic symptoms are not as severe. In general, in clinical practice it is difficult to discern between positive symptoms of SSDs and autism symptoms. For example, sensory issues present in individuals with autism may be misdiagnosed as hallucinations in schizophrenia with significant treatment implications. Similarly, it can be also difficult to discern between negative symptoms of schizophrenia and autism symptoms. For example, the lack of emotional reciprocity that is present in individuals with autism resemble the blunt affect or alogia (i.e., poverty of speech) in schizophrenia. Therefore, it is possible that individuals with SSDs develop autistic-like symptoms as a result of their negative symptoms. Our results are consistent with previous research in this area reporting that autistic symptoms seem to be prevalent in SSDs, ranging from 9.6 to 61% (6).

However, the findings of this meta-analysis should be interpreted with some caution. Limitations include the paucity of studies analyzed, different diagnostic criteria and measures, and differences in the age of the samples.

In our meta-analysis, we included only 13 studies. The pool of 13 studies was small and did not allow meta-regression analyses to examine how participants characteristics (e.g., gender, age, illness duration) can predict autistic symptoms. However, the total number of participants included in the current meta-analysis is large (*n* = 1,958) and the results are strong, with high SMDs, and precise 95%CIs.

Furthermore, we found studies with different diagnostic criteria, which is a reason for increased heterogeneity. However, it has to be noted that all the diagnostic criteria used by studies included on the current meta-analysis were well-validated and all studies used either a DSM based diagnosis, or the Structured Interview for Prodromal Symptoms (SIPS), or the Positive and Negative Syndrome Scale (PANSS) for the diagnosis of SSDs. Indeed, the AQ is a self-report scale, which may introduce reporting bias. Moreover, this measure was not always available, and we needed to use other instruments measuring autistic symptoms. Therefore, given the different scales used in the current meta-analysis, we analyzed data by SMDs as opposed to mean differences.

Three studies included only children and adolescents (18, 38, 39), which may differ from adults in terms of diagnosis stability and autistic symptoms. Therefore, we decided to undertake subgroup analyses taking into account the age. ASD by definition is a disorder present from early childhood. It is important to consider the individual's developmental history in order to distinguish between the presence of an ASD and autistic symptoms. However, it is still unclear whether difficulties such as those present in individuals with autism are present before the onset of schizophrenia or whether they are dependent by the schizophrenic state itself (e.g., resulting from thought dysfunction), thus not truly autistic.

Further studies investigating the etiology of autism symptoms in children and adolescents with SSDs are needed in order to shed light on this issue. Furthermore, longitudinal studies investigating autistic symptoms in high-ultra risk state populations can clarify whether these symptoms are stable features of this clinical population or are dependent by the schizophrenic state of illness. Indeed, it has to be noted that in the present meta-analysis only one study (41) used a diagnostic tool for autism (i.e., ADOS-2). All other studies used instruments that were designed as screening measures for autism. Therefore, in order to distinguish between trait and state, future studies would benefit by the use of diagnostic measures (e.g., ADOS-2) along with developmental history in order to confirm whether any deficit associated with autism was present before the onset of SSDs. Indeed, it is well-known that individual with autism are at an increased risk to develop other mental-health conditions and a ASD diagnosis is particularly difficult in adults, especially when knowledge of early developmental history is missing. Therefore, longitudinal studies investigating autistic symptoms in individuals with SSDs in different psychopathological phases could clarify whether these characteristics persist after recovery from SSDs.

## Conclusion

In conclusion, we found that individuals with SSDs have higher autistic symptoms than healthy controls and lower autistic symptoms than individuals with autism. To our knowledge, this is the first systematic review and meta-analysis trying to quantitatively pool all evidences on the topic. Our study has some limitations, including the use of self-report scales, which may introduce reporting bias. Therefore, further studies investigating the etiology of autism symptoms are needed to shed light on the association between these conditions.

## Author Contributions

MS and VP overviewed and examined the literature. FD performed the statistical analysis. FD, VP, AR, and MS wrote the manuscript. LM, MA, and PC designed the study, contributed to theoretical interpretation read and final proof reading. Each author read and approved the final version of the manuscript.

### Conflict of Interest Statement

The authors declare that the research was conducted in the absence of any commercial or financial relationships that could be construed as a potential conflict of interest.
